# Beyond Repetition: The Role of Gray Zone Alleles in the Upregulation of *FMR1*-Binding miR-323a-3p and the Modification of BMP/SMAD-Pathway Gene Expression in Human Granulosa Cells

**DOI:** 10.3390/ijms26073192

**Published:** 2025-03-29

**Authors:** Adriana Vilkaite, Xuan Phuoc Nguyen, Cansu Türkan Güzel, Lucas Gottschlich, Ulrike Bender, Jens E. Dietrich, Katrin Hinderhofer, Thomas Strowitzki, Julia Rehnitz

**Affiliations:** 1 Department of Gynecological Endocrinology and Fertility Disorders, University Women’s Hospital, 69120 Heidelberg, Germany; adriana.vilkaite@gmail.com (A.V.); phuocxp@gmail.com (X.P.N.); tuerkan.guezel@med.uni-heidelberg.de (C.T.G.); lucas.gottschlich@med.uni-heidelberg.de (L.G.); ulrike.bender@med.uni-heidelberg.de (U.B.); jens.dietrich@med.uni-heidelberg.de (J.E.D.); thomas.strowitzki@med.uni-heidelberg.de (T.S.); 2 Institute of Human Genetics, University Heidelberg, 69120 Heidelberg, Germany; katrin.hinderhofer@med.uni-heidelberg.de

**Keywords:** human granulosa cells, FMR1, ovarian function, miR-323a-3p, SMAD

## Abstract

The Fragile X mental retardation type 1 gene (*FMR1*) contains a CGG triplet cluster of varied length (30 repeats on average) located in its 5′ UTR. In its premutated state (54–200 repeats), *FMR1* contributes to the pathogenesis of premature ovarian insufficiency (POI). Its gray zone alleles (41–54 repeats) are supposed to impair the ovarian function as well. In the case of a CGG repeat length > 200, Fragile X syndrome occurs. Post-transcriptional expression of *FMR1* is regulated by microRNAs. Although miR-323a-3p overexpression suppresses *FMR1* in various tissues, this relationship has not been evaluated in the human ovary. Additionally, this microRNA targets *SMAD*s, which are suggested regulators of ovarian cell proliferation, growth, and function. This study investigated how *FMR1* allele lengths with CGG repeat numbers *n* < 55 (normal and gray zone genotypes) relate to miR-323a-3p expression and how they may impact associated *SMAD* expression in human granulosa cells. COV434 cells and patient-derived GCs were used to evaluate *FMR1*, miR-323a-3p, and BMP/SMAD-pathway member expression levels. Briefly, miR-323a-3p was significantly upregulated in GCs of the gray zone group compared to the normal allele group (*p* < 0.0001), while the *FMR1* level did not vary. Furthermore, the gray zone group showed a significant upregulation of *BMPR2*, *SMAD1*, *SMAD4*, and *SMAD9*. In contrast, the miR-323a-3p transfection of COV434 cells significantly downregulated *SMAD3*, *SMAD4*, *SMAD5*, and *SMAD9*, while the *FMR1* and *SMAD1* levels remained stable. Our findings highlight a CGG repeat number-dependent upregulation of miR-323a-3p and an alteration of the BMP/SMAD pathway, suggesting that these changes happen and contribute to impaired ovarian function independently.

## 1. Introduction

One of the most critical factors for successful reproduction is the presence of well-regulated ovarian follicular maturation, known as folliculogenesis. Disorders affecting this mechanism may lead to a diminished ovarian reserve or premature ovarian insufficiency (POI), resulting in difficulties for patients who, for example, are undergoing assisted reproductive treatments [1]. Approximately 1% of all biological women are suffering from POI due to various reasons [2]. Approximately 9–24% of patients who undergo in vitro fertilization (IVF) exhibit a poor response to standard ovarian stimulation protocols [3], often leading to reduced oocyte quality and low pregnancy rates.

*FMR1*, located on the distal part of the long arm of the X chromosome, together with the FMRP protein, expressed mainly in neural and germ cells, is a primary regulator of folliculogenesis and one of the most common genetic causes underlying ovarian insufficiency [4,5]. Fragile X-associated POI (FXPOI) is caused by the expansion of a region in the 5′ untranslated region (UTR) of *FMR1*, which includes a cluster of CGG trinucleotide repeats [6]. When a fully mutated *FMR1* gene (>200 CGG repeats) is present, intellectual disability is observed; however, it does not affect female ovarian function [4,7]. FXPOI is observed in approximately 20% of premutation carriers exhibiting a CGG length of 55–200 repeats [4]. While the normal CGG triplet repeat number can be described as lower than 41, the intermediate or so-called gray zone range is defined as a repeat number between 41 and 54 [8]. Its rate in the general population ranges from 0.8% to 3.0% [9]. These alleles do not exhibit premutation-typical RNA toxicity [10]. However, the gray zone alleles display potential transmissional instability, with the possibility of further expansion in future generations [11]. In addition to altered *FMR1* mRNA levels [12], evidence suggests a possible connection between gray zone alleles and poor ovarian response (POR), as carriers may present with lower levels of ovarian reserve markers (antral follicle count or anti-Müllerian hormone) [13,14]. The correlation between normal CGG triplet repeats (<41) and ovarian function remains undetermined since certain women with normal CGG triplet repeats exhibit POR as well [5,13,15].

*FMR1* codes for FMRP (Fragile X mental retardation type 1 protein), which is primarily expressed in neural and germ cells [16,17]. FMRP, which functions as a translational inhibitor and is expressed in the oocyte as well as granulosa cells (GCs) in the ovaries of women of various age groups, is assumed to play a critical role in controlling gametogenesis. Although the exact mechanisms have not been discovered yet, it is suggested that FMRP controls germline cell proliferation, differentiation, and maintenance [18,19,20,21,22,23].

Folliculogenesis and ovarian function are complex and require finely tuned regulation. Evidence suggests that microRNAs are crucial in regulating human GCs [24]. MicroRNAs are highly conserved endogenous non-coding RNAs, approximately 18–25 nucleotides long [25]. They participate in the post-transcriptional regulation of gene expression by binding to the 3′ UTRs of their target mRNAs, resulting in translational inhibition and/or gene silencing [26]. Studies have revealed that disrupted miRNA maturation affects somatic GCs, leading to total female sterility because of morphological changes and impaired ovarian function [27,28,29]. Further studies have acknowledged that microRNAs regulate GC pathophysiology in different ways, e.g., via follicle-stimulating hormone (FSH), transforming growth factor-β (TGF-β), and other hormone-related and apoptotic pathways [30]. Moreover, *FMR1* is among the first genes identified to be linked to microRNA pathways [31], suggesting that microRNAs play a pivotal role in the development of *FMR1*-associated reproductive disorders, including FXPOI and POR [32,33,34].

One example of an *FMR1*-targeting microRNA is miR-323a-3p, which performs a tumor-suppressor function in various cancers by targeting *FMR1* and suppressing its levels [34,35,36,37]. Despite robust evidence supporting this relationship, no studies have yet explored the interaction between overexpressed miR-323a-3p and *FMR1* in the female ovary. Furthermore, besides *FMR1*, a search using the TargetScan (https://www.targetscan.org) and MIRDB (https://mirdb.org) databases revealed that miR-323a-3p, previously known as miR-323-3p, also targets SMAD pathway members, namely *SMAD2*, *SMAD3*, and *SMAD5*. SMADs belong to the TGF-β superfamily of ligands, and their signaling pathways, once activated by oocyte-secreted factors, play a crucial role in controlling cell proliferation, differentiation, and apoptosis in various organ systems, including the human ovaries [38]. One activator of the SMAD pathway is bone morphogenetic protein (BMP), which, after binding to the bone morphogenetic protein receptor type II (BMPR2), phosphorylates SMADs and mediates GC function and follicular development [39]. Specifically, the SMAD2/3 pathway regulates folliculogenesis, ovulation, and cumulus cell growth [40,41,42,43], whereas the SMAD1/5/9 signaling cascade controls essential biological functions of the ovary, including primordial follicle activation, GC and theca cell proliferation, metabolism, steroidogenesis, and oocyte development [44,45,46,47]. In addition, SMAD proteins can interact with microRNAs, potentially modulating follicular atresia and influencing female reproduction [48,49,50].

The primary objective of this study was to investigate the impact of gray zone *FMR1* allele length (41–54 CGG repeats) on miR-323a-3p and the SMAD pathway gene expression levels in female germline cells with regard to *FMR1* expression and different ovarian responses to hormonal stimulation during ART. The emphasis was set on assessing the impact of the presence of at least one gray zone allele on miR-323a-3p, *FMR1*, and *SMAD* expression in GCs, both ex vivo and in vitro, as well as on investigating whether the expression of miR-323a-3p and *FMR1* differs between poor and normal ovarian responders. Understanding these interactions may provide valuable insight into the molecular mechanisms underlying diminished ovarian reserve.

## 2. Results

### 2.1. Patient Demographics

A total of 83 patients participated in this study, with 55 presenting with a normal genotype and 28 presenting with gray zone alleles (Table 1). No significant difference was found between the two groups regarding patient age, BMI, FSH, LH, estradiol and AMH levels, antral follicle count, total retrieved oocytes, and mature oocytes.

### 2.2. FMR1 Expression Is Independent of Gray Zone Allele Presence

**Figure 1:**
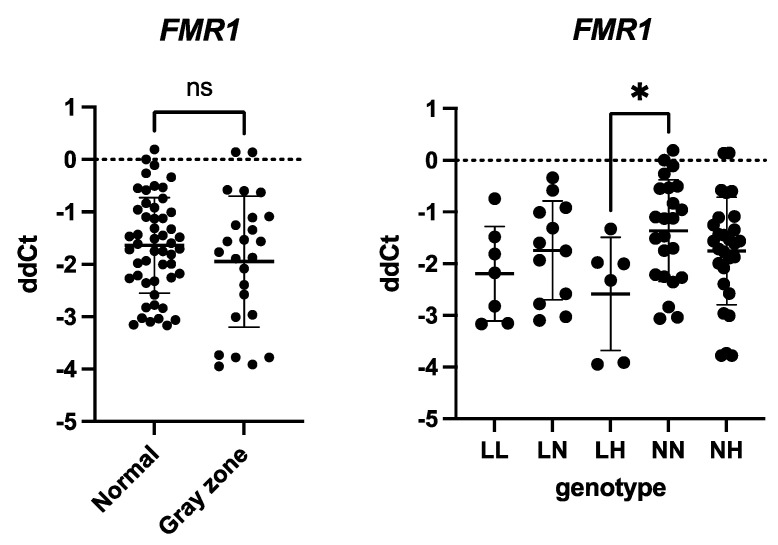
*FMR1* expression in human granulosa cells. mRNA levels were analyzed using TaqMan based on real-time polymerase chain reaction. *FMR1* mRNA levels were normalized to *TBP* and *HPRT1* mRNA expression, and the results are presented as comparative Ct (ddCt) values (means ± SD; performed in triplicates). Statistical significance classification: ns: *p* > 0.05; *: *p* ≤ 0.05. *FMR1* expression was stable between normal and gray zone genotype groups, *p* = 0.2218 (unpaired Student’s *t*-test); *FMR1* mRNA was significantly upregulated in the LH genotype group compared to the NN genotype group, set as the control group, *p* = 0.0370 (ordinary one-way ANOVA).

We initially aimed to evaluate the expression of *FMR1* in the patients’ granulosa cells to compare its expression levels in normal and gray zone genotypes (*n_Nor_* = 55, *n_Gray_* = 28). Thus, we performed RT-qPCR. Our results indicated a stable level of *FMR1* in both groups (*p* = 0.2218, Figure 1). We additionally performed a genotype analysis (*n_LL_* = 7, *n_LN_* = 12, *n_LH_* = 6, *n_NN_* = 23, *n_NH_* = 28; abbreviations defined in Methods Section 4.2). It showed a statistically significant upregulation of *FMR1* expression in the low–high genotype group compared to the normal–normal genotype group (*p* = 0.0370, Figure 1).

### 2.3. miR-323a-3p Expression Significantly Increased in GCs of the Gray Zone Group

**Figure 2:**
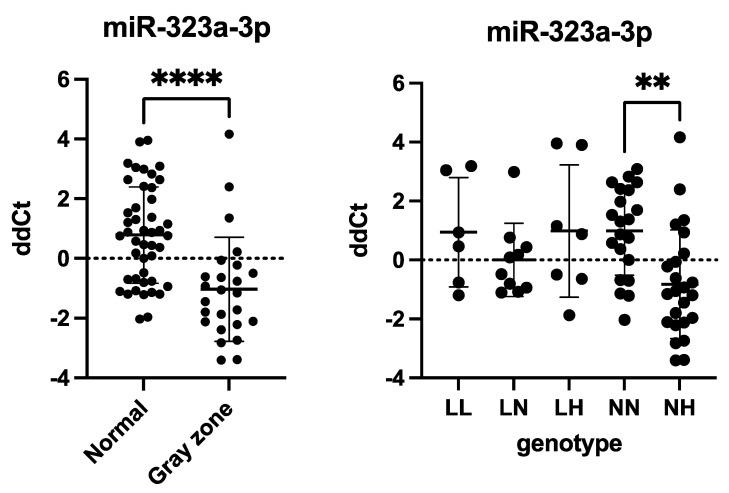
miR-323a-3p expression in human granulosa cells. microRNA levels were analyzed using TaqMan-based real-time polymerase chain reaction. miRNA levels were normalized to miR-16 expression, and the results are presented as comparative Ct (ddCt) values (means ± SD; performed in triplicates). Statistical significance classification: **: *p* ≤ 0.01; ****: *p* ≤ 0.0001. miR-323a-3p expression was significantly upregulated in granulosa cells of gray zone patients, *p* < 0.0001 (unpaired Student’s *t*-test); miR-323a-3p was significantly upregulated in the NH genotype group compared to the NN genotype group, set as the control group, *p* = 0.0031 (ordinary one-way ANOVA).

To investigate whether miR-323a-3p plays a role in ovarian function, we analyzed its levels in GCs (*n_Nor_* = 45, *n_Gray_* = 25). We quantified miR-323a-3p levels using RT-qPCR by extracting RNA and transcribing it into microRNA-specific cDNA. Our results revealed a significant upregulation of miR-323a-3p in the gray zone group compared to the normal genotype group (*p* < 0.0001, Figure 2), illustrated by the lower ddCt values. A separate genotype analysis (*n_LL_* = 6, *n_LN_* = 10, *n_LH_* = 7, *n_NN_* = 21, *n_NH_* = 24) showed a statistically significant upregulation of miR-323a-3p expression in the normal–high genotype group compared to the normal–normal genotype group (*p* = 0.0031, Figure 2).

### 2.4. FMR1 Levels Were Downregulated in Poor Ovarian Responders

**Figure 3:**
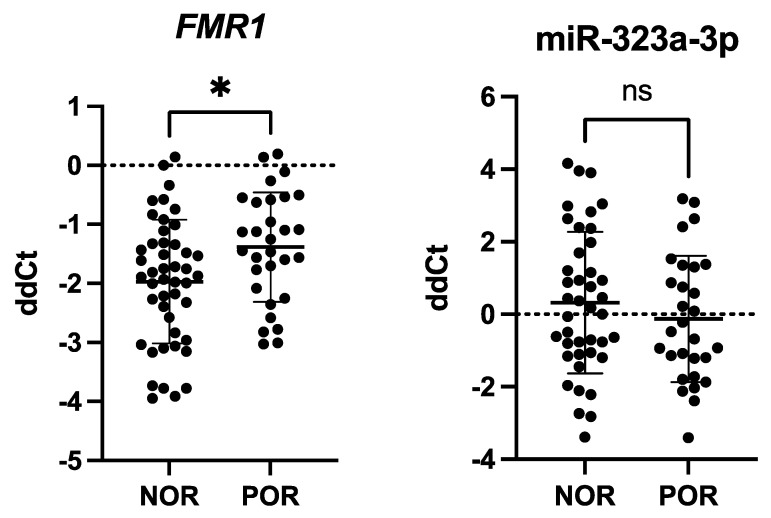
*FMR1* and miR-323a-3p expression in human granulosa cells. mRNA and microRNA levels were analyzed using TaqMan-based real-time polymerase chain reaction. *FMR1* mRNA levels were normalized to *TBP* and *HPRT1* mRNA expression. miRNA levels were normalized to miR-16 expression. The results are presented as comparative Ct (fold change) values (means ± SD; performed in triplicates). Statistical significance classification: ns: *p* > 0.05; *: *p* ≤ 0.05. *FMR1* expression was downregulated in granulosa cells of patients with poor ovarian response, *p* = 0.0145 (unpaired Student’s *t*-test); miR-323a-3p expression remained stable, *p* = 0.3191 (unpaired Student’s *t*-test).

In order to evaluate the relationship between ovarian response and *FMR1* and miR-323a-3p expression, we performed comparative analyses of normal (NOR) and poor (POR) ovarian responders. In total, 30 women were assigned to the POR group based on the Bologna Criteria (Ferraretti et al. 2011) [51]. Of them, 11 were gray zone carriers. The remaining 46 participants, including 18 gray zone patients, were classified as having a normal ovarian response (NOR) and constituted the control group. We found a significant downregulation of *FMR1* mRNA in the POR group compared to the NOR group (*p* = 0.0145), while the miR-323a-3p levels remained stable (Figure 3). An additional contingency analysis with the χ^2^-test did not show a link between gray zone allele carriers and poor ovarian response (*p* = 0.8565).

### 2.5. miR-323a-3p Overexpression Decreased SMAD Levels in COV434 Cells

To explore whether alterations in miR-323a-3p expression levels affected the SMAD pathway, we cultured the COV434 cell line and transfected the cells with a predesigned miR-323a-3p mimic, whereas a nonspecific treatment (“scramble”) was used as a negative control. Initially, we compared cell growth after 24 and 48 h of transfection and found that the shorter incubation time resulted in good cell viability, with cell numbers increasing approximately three-fold. Conversely, after 48 h, most of the cells had undergone apoptosis. Subsequently, we transfected the cells with treatments at three different concentrations (10, 30, and 50 nM) to determine the most effective one. All the treatments increased miR-323a-3p expression in COV434 cells, but the 50 nM concentration displayed the highest efficiency, with miR-323a-3p levels increasing by 145-fold (Supplementary Figure S1).

**Figure 4:**
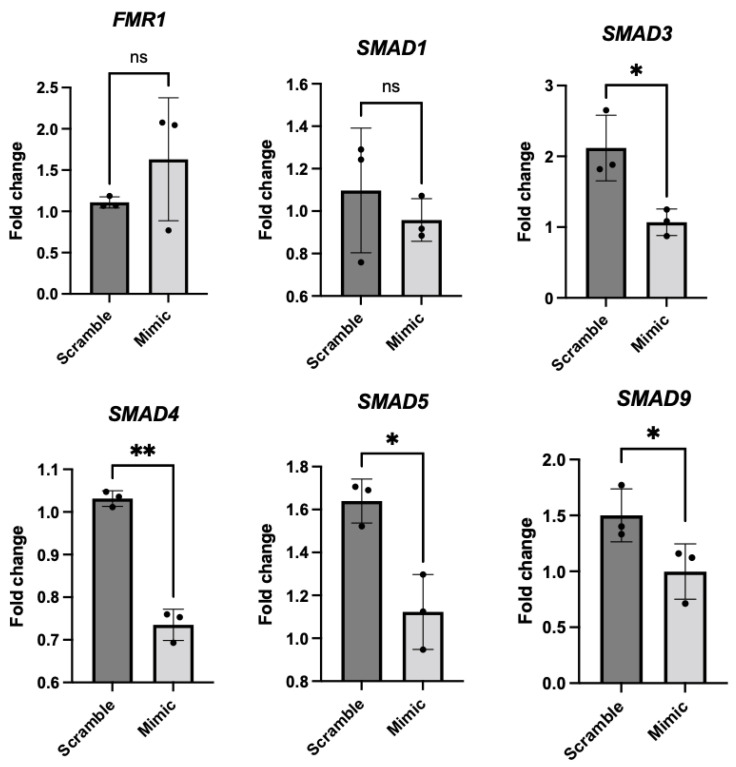
*FMR1*, *SMAD1*, *SMAD3*, *SMAD4*, *SMAD5*, and *SMAD9* expression in COV434 cells after miR-323a-3p mimic treatment. Gene mRNA levels in COV434 cells, treated with scramble and mimic for 24 h, were analyzed using TaqMan-based real-time polymerase chain reaction. mRNA levels were normalized to *TBP* and *HPRT1* expression, and the results are presented as comparative Ct (fold change) values (means ± SD; performed in triplicates). Statistical significance classification: ns: *p* > 0.05; *: *p* ≤ 0.05; **: *p* ≤ 0.01. The *p*-values are as follows: *FMR1*, 0.33; *SMAD1*, 0.44; *SMAD3*, 0.029; *SMAD4*, 0.0068; *SMAD5*, 0.0448; *SMAD9*, 0.047 (paired Student’s *t*-test).

We subsequently analyzed the gene expression in COV434 cells transfected with 50 nM of miRNA-323a-3p mimic. The RT-qPCR results revealed no significant changes in *FMR1* and *SMAD1* expression (*p* = 0.33 and *p* = 0.44, respectively), and significant downregulation of *SMAD3*, *SMAD4*, *SMAD5,* and *SMAD9* (*p* = 0.029, *p* = 0.007, *p* = 0.045, and *p* = 0.047), indicated by lower fold change values, was observed (Figure 4).

### 2.6. BMPR2 and SMADs Were Upregulated in GCs of the Gray Zone Group

**Figure 5:**
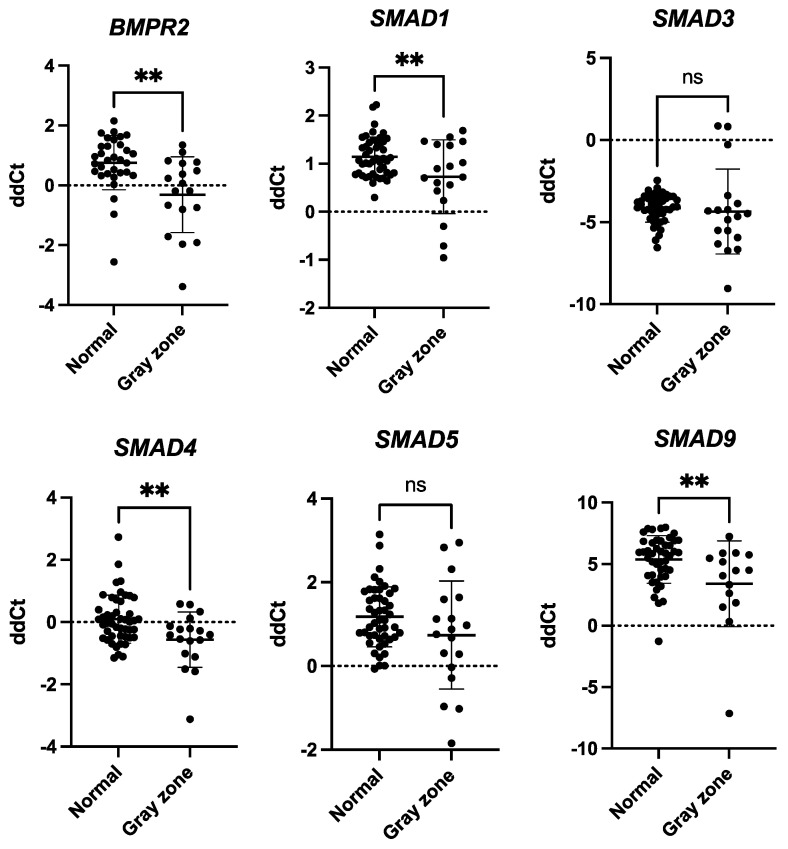
*SMAD1*, *SMAD3*, *SMAD4*, *SMAD5*, *SMAD9*, and *BMPR2* expression in human granulosa cells. mRNA levels were analyzed using TaqMan-based real-time polymerase chain reaction. *BMPR2*, *SMAD1*, *SMAD3*, *SMAD4*, *SMAD5,* and *SMAD9* mRNA levels were normalized to *TBP* and *HPRT1* mRNA expression, and the results are presented as comparative Ct values (means ± SD; performed in triplicates). Statistical significance classification: ns: *p* > 0.05; **: *p* ≤ 0.01. *SMAD3* and *SMAD5* did not display differences in expression between normal and gray zone genotypes, *p*-values: 0.6240 and 0.0874, respectively (unpaired Student’s *t*-test). *BMPR2*, *SMAD1*, *SMAD4*, and *SMAD9* expressions were significantly upregulated in GCs of patients with gray zone genotype compared to normal genotype, *p*-values: 0.0011, 0.0067, 0.0046, and 0.0077, respectively (unpaired Student’s *t*-test).

To explore whether our in vitro findings were reflected in the study population, we further analyzed ex vivo samples from women with different genotypes. Specifically, we evaluated the levels of *BMPR2* and *SMAD*s in GC samples (*n_Nor_* = 47, *n_Gray_* = 18). The goal was to determine whether the trends observed in our in vitro experiments were consistent with the expression patterns observed in the patients’ GCs. We found *BMPR2* to be upregulated in the gray zone patients (*p* = 0.0011, Figure 5). Among the *SMAD*s analyzed, the *SMAD3* and *SMAD5* levels remained stable (*p*-values: 0.6240 and 0.0874, respectively, Figure 5), while *SMAD1*, *SMAD4*, and *SMAD9* exhibited a significant difference in relative expression between the two groups. In particular, their expression was upregulated in GCs from patients with gray zone alleles compared to that in GCs from patients with normal genotypes (*p* = 0.0067, *p* = 0.0046, *p* = 0.0077, respectively, Figure 5).

## 3. Discussion

**Figure 6:**
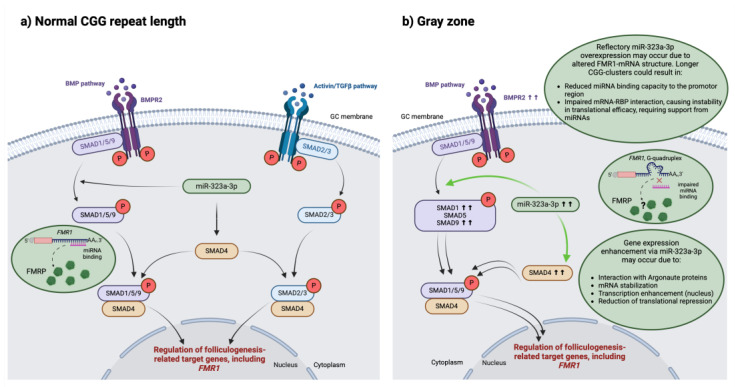
Changes in miR-323a-3p and BMP/SMAD pathway in gray zone carriers: proposed mechanism of action. Our study highlights changes in regulatory mechanisms of granulosa cells in *FMR1* gray zone allele carriers, potentially interrupting the regulation of folliculogenesis. Arrows indicate direction of molecular interaction. Firstly, the observed overexpression of miR-323a-3p may reflectorily occur due to structural changes in *FMR1* mRNA. Longer CGG triplet repeat cluster zones could result in a more complex structure of the mRNA, such as the formation of G-quadruplexes, ultimately reducing miRNA binding capacity to the promotor region or impairing mRNA/RBP interaction, thus reducing translational efficiency and requiring microRNA-mediated assistance. Secondly, the observed increase in *BMPR2* and *SMAD*s could directly result from increased miR-323a-3p. Overexpressed miR-323a-3p may trigger gene transcription enhancement (green arrows indicate increased SMAD transcription via miR-323a-3p) via several mechanisms, elucidated in the figure, and ultimately affect the extent to which BMP/SMAD pathway members execute their functions, such as regulating cell proliferation, steroidogenesis, and follicle and oocyte development (double arrows indicate increased functional activity). The figure was created using BioRender (https://www.biorender.com, accessed on 28 January 2025).

In this study, we investigated the importance of *FMR1*-associated miR-323a-3p in the context of ovarian function regulation. Our main findings provide insight into the involvement of miR-323a-3p and its downstream effects on BMP/SMAD signaling in the so-called gray zone allele carrier granulosa cells. Our proposed regulatory mechanism is schematically illustrated in Figure 6 and further discussed at the end of this section.

Folliculogenesis, the dynamic process of follicle development, is finely tuned by multiple molecular mechanisms [52,53]. One of the most prominent regulators of folliculogenesis and a common genetic cause of ovarian insufficiency is *FMR1* [4,5]. In FXPOI, the phenotype originates from the expansion of a CGG triplet repeat region in the 5′ UTR of premutated *FMR1* [6]. This ovarian insufficiency affects around 20% of the premutation carriers, and the CGG triple repeat length ranges from 55 to 200 [4]. The relationship between gray zone (41–54) CGG triplet repeat lengths, observed in 0.8–3.0% of the general population, and ovarian function remains unclear [9]. A 2014 study found lower AMH levels in women with normal–high and gray zone CGG repeat numbers (35–50) compared to those with numbers below 35, implicating a CGG repeat-dependent reduction in ovarian reserve even before the diagnosis of premature ovarian insufficiency [54]. Lledo et al. investigated the association of different allele lengths with ovarian response. They found no negative effect on response to ovarian stimulation for normal and intermediate length alleles [55], while others reported an association between premature ovarian failure and increased occurrence of gray zone *FMR1* alleles [13,15]. Interestingly, our patient cohort did not demonstrate any difference between reproductive parameters. In particular, the patient AMH levels in both normal and intermediate-range allele carriers were similar. Considering that AMH is suggested to be one of the best markers for ovarian reserve decline in otherwise healthy women [56], the results of our study imply no direct link between gray zone carriers and diminished ovarian reserve. Additionally, we showed the stability of *FMR1* levels among the normal and gray zone groups, contrary to a previous publication, which displayed an increased *FMR1* mRNA expression in gray zone allele carriers [12]; this may be due to the 2007 study being conducted exclusively on male subjects, while our research dealt with female patient samples. It is important to note that a separate genotype analysis, including all CGG repeat numbers below the premutation range (<55), revealed an upregulation of *FMR1* mRNA in the low–high group (one allele < 26 and the other 35–55 compared to the normal–normal group; both alleles fall in the range of 26–34). The discrepancies between the current and previously conducted research prove that the current knowledge of the intricacies of transcriptional changes in different lengths of the CGG cluster is sparse, and further investigation is needed.

In the past decade, research has revealed the significance of miRNAs in ovarian development and function [57]. miRNAs play critical roles in primordial follicle formation, recruitment, selection, follicular atresia, oocyte–cumulus cell interaction, GC function, and luteinization [57,58,59]. Furthermore, miR-323a-3p is reportedly correlated with regulating cell proliferation, apoptosis, and differentiation in various tissues and diseases [60,61]. The overexpression of miR-323-3p in human brain gliomas and neuronal cells inhibits cell proliferation and induces apoptosis [62,63]. In ovarian biology, miR-323 has been reported to regulate GC functions, including cell proliferation, apoptosis, and steroidogenesis, which are critical for appropriate follicle development [64]. Increased miR-323 expression was noted in GCs from patients with polycystic ovary syndrome, characterized by impaired follicle maturation and abnormal hormone production [65]. This evidence suggests a potential role for miR-323a-3p in modulating crucial signaling pathways involved in folliculogenesis. Research has demonstrated that miR-323a-3p plays a regulatory role in the expression of *FMR1* and its coding protein, FMRP [34,35]. Specifically, miR-323a-3p directly targets the 3′ UTR of *FMR1* mRNA, resulting in the downregulation of *FMR1* expression [35]. *FMR1* is a crucial gene involved in Fragile X syndrome, a genetic disorder associated with intellectual disability and FXPOI [66,67]. FMR1 and FMRP have also been implicated in GC functions and oocyte maturation [68,69]. FMRP interacts with several mRNAs, including those encoding proteins that play vital roles during folliculogenesis, such as cell cycle regulation and translation [6,69,70,71]. Consequently, changes in the expression of *FMR1* and FMRP can disrupt these molecular signaling pathways, impacting follicular development, maturation, and ovulation [5,70,71]. To the best of our knowledge, the current study is the first to report very strongly increased miR-323a-3p levels in granulosa cells from women with gray zone alleles relative to those in women with normal CGG repeat numbers, suggesting that miR-323a-3p-mediated effects may play a pivotal role in folliculogenesis and ovarian function. However, a conclusion specifically elucidating the exact nature of the consequences of increased miR-323a-3p expression for granulosa cells, such as an altered proliferation index or apoptosis rate, cannot yet be drawn due to our study primarily focusing on gene expression and should be the main focus of our future studies.

Alterations in *FMR1* expression have been suggested to influence patient responsiveness to ovarian stimulation in ART treatments [43,72]. In this study, we explored the level of *FMR1* in relation to ovarian response and its potential association with miRNA-323a-3p dysregulation. We observed the downregulation of *FMR1* in patients with POR, while the miRNA-323a-3p level remained stable. Even though this microRNA may not be directly linked to poor ovarian response, the relationship between miRNAs and their target genes, including *FMR1,* is complex and involves intricate regulatory mechanisms, such as post-transcriptional modifications and feedback loops. Further studies with larger sample sizes are necessary to elucidate the specific interactions between miRNA-323a-3p and *FMR1*.

miRNAs possess the ability to repress multiple target genes [73]. Our examination of the TargetScan and MIRDB databases revealed that miRNA-323a-3p, previously miR-323-3p, targets various TGF-β signaling pathway components, including *SMAD2*, *SMAD3*, *SMAD4*, *SMAD5*, and *SMAD9*. Earlier studies demonstrated that miRNA-323-3p inhibits the expression of SMAD family members, such as *SMAD2* and *SMAD3*, by directly interacting with their 3′ UTRs [60,74,75] at the gene level. SMADs function downstream in the TGF-β signaling pathway [76], critically regulating GC activities, follicular growth, and oocyte maturation [77,78]. TGF-β ligands, including TGF-β1, activins, and inhibins, are secreted by oocytes, GCs, and theca cells in ovarian follicles [79]. Ligand activation triggers a signaling cascade that ultimately leads to the activation of SMAD proteins. One such ligand is BMP, a member of the TGF-β superfamily, the main ovarian function of which is mediating apoptosis and follicular development [39]. In GCs, the activation of BMPR2 via BMP results in the phosphorylation of *SMAD1*, *SMAD5*, and *SMAD9*. Once activated, SMADs translocate to the nucleus and regulate the expression of target genes involved in GC proliferation, differentiation, and steroidogenesis [42,53,76,79]. The dysregulation of the SMAD pathway can disrupt these critical processes, resulting in reproductive disorders associated with impaired folliculogenesis [42].

Our in vitro experiments provided the first evidence of the suppression of *SMAD3*, *SMAD4*, *SMAD5*, and *SMAD9* mRNA expression by miR-323a-3p in human granulosa cells of the COV434 cell line. We decided to perform the functional tests on this granulosa cell line because fresh granulosa cells of distinct amounts are restricted in availability and show a short lifespan, which would have adversely affected the performance of our functional tests. Fortunately, the COV434 cell line, although of tumorous origin, demonstrates multiple properties and specialties typical for natural granulosa cells [80]. It is important to note, however, that, in our research, COV434 serves as an in vitro model only, and it cannot be guaranteed that these cells, derived from a granulosa cell tumor, are identical in their qualities to the GCs retrieved directly from patients’ ovaries. While it is possible that conducting the same experiments on cultivated fresh granulosa cells would have produced different results, this, too, comes with limitations. Primary cultures are difficult to cultivate with sufficient proliferation and division for functional analyses. Additionally, primary cultures always reflect the specific donor’s physiology, introducing a donor-specific variability bias; this contrasts with the data obtained from a well-established cell line, which offers greater reproducibility. Primary cell culture would require multiple independent cultivations from individuals with different ovarian physiology to validate and generalize the findings. However, that would, in return, reduce the reproducibility and comparability of this functional model, as inter-individual variability could obscure the underlying molecular mechanisms.

The downregulation of *SMAD*s observed in our study is consistent with previous findings [74,75], suggesting that dysregulated SMAD signaling can affect normal follicular development and impair ovarian response. In particular, the clear upregulation of *SMAD1*, *SMAD4*, and *SMAD9* in GCs of gray zone allele carriers indicates that the in vivo regulatory pathways and molecule interactions differ from those observed in our in vitro model. One possible explanation for this difference could be the cancer origin of the COV434 cell line. In a 2017 study with bladder cancer cells performed by Li et al., a transfection of two separate bladder cancer cell lines with an miR-323a-3p mimic resulted in a suppression of SMAD3, ultimately showing miR-323a-3p-mediated repression of tumor progression [36]. Similar effects have been observed in neuroblastoma and colorectal cancer cell lines [81,82]. In summary, in cancer cell lines, upregulation of miR-323a-3p caused suppression of *SMAD* family members, which is also observed in the in vitro part of the present study. However, a strong upregulation of miR-323a-3p in healthy donor granulosa cells, comparable to a mimic transfection of cell culture, unexpectedly concurred with increased *SMAD* expression. As mentioned before, different cell origins could be speculated as a reason for this discrepancy. Alternatively, regulating the SMAD pathway in gray zone allele carriers may also occur independently of miR-323a-3p.

Our group has previously shown that suppression of *FMR1* in vitro reduced the level of FMRP and increased the level of BMPR2, both on mRNA and protein levels, in COV434 cells as well as showed downregulation of *FMR1* and *BMPR2* mRNA in poor ovarian responders compared to patients with a normal response to ovarian stimulation [43]. Interestingly, the current results showcase an upregulation of *BMPR2* in carriers of gray zone alleles without alterations in *FMR1* levels, suggesting that the effects of this allele are mediated through different regulatory mechanisms than those of poor ovarian response. It is important to note that mRNA expression changes alone do not always correspond to changes of the same extent in protein expression [83]. As reviewed in [84], up to 85% of protein level variation could be attributed to alterations in mRNA levels. In eukaryotes, transcription is not the only process contributing to translational efficiency. An impactful factor prior to translation is, for example, the length of poly (A) tails of mRNA or phosphorylation of regulatory elements, such as RNA-binding proteins or microRNA. Additionally, cellular protein levels are regulated by the processes of degradation and proteolysis, which are not directly related to transcriptional efficiency [84]. However, despite these factors, significant differences in mRNA expression are commonly considered functionally relevant due to the well-established, reliable methods of mRNA quantification and relatively good sample availability [83]. In premutation patients, increasing *FMR1* mRNA corresponds with decreasing FMRP levels [85], but this relationship has not been evaluated in the gray zone range. While we did not observe changes in FMR1 mRNA in gray zone patients in our current study, evaluating the protein level would provide more definite conclusions. Likewise, our observed decrease in *FMR1* in poor ovarian response should be prospectively followed-up by a protein expression evaluation. Our group has previously shown that an *FMR1*-silencing-mediated increase in *BMPR2* mRNA in COV434 cells corresponds with an increase in the BMPR2 protein [43], allowing for the assumption that our presently observed elevated mRNA levels in gray zone patient samples would also have a similar effect on the protein level. However, due to various post-translational regulatory mechanisms [86], it is not possible to anticipate the exact changes in SMAD family member protein expression on mRNA levels only. Since this study focused only on mRNA, not on protein expression levels, this limitation should be addressed by further studies investigating the BMP/SMAD pathway member expression patterns and the influence *FMR1*/FMRP and miR-323a-3p exert on it at the protein level.

We summarize our findings in the graph depicted in Figure 6. While our results display clear expression level changes in gray zone allele carriers at the mRNA level, the exact localization of the occurring disruption cannot be determined from this study alone. We propose several possible reasons for our observed alterations in gray zone allele carriers. The key findings of this study are elevated levels of miR-323a-3p and BMP/SMAD pathway members. miR-323a-3p is an *FMR1*-binding microRNA [35]. Differences in *FMR1*/FMRP structure and activity could result in subsequent adjustments of miR-323a-3p expression level. In *FMR1* mRNA, the CGG repeat cluster forms a hairpin structure [87,88]. In premutation cases, defined by CGG triplet numbers in the range of 55–200, this structure becomes more complex, contributing to mRNA toxicity [88,89]. It is possible that, already in gray zone alleles, some structural changes start occurring, reducing miR-323a-3p’s capacity to bind to the 3′UTR of *FMR1* mRNA or causing mild cellular toxicity, as suggested in a 2020 study by Hall et al., which found gray zone alleles to be associated with parkinsonism and increased mortality [90]. Previous research discussed structural changes in mRNA and their impact on microRNA binding. For example, sequences rich in guanine, such as the CGG repeat cluster in *FMR1*, tend to form toxic G-quadruplexes, and this formation has also been shown to inhibit successful interaction between microRNAs and their target mRNAs [91,92,93]. Additionally, altered mRNA structure could impair *FMR1* interaction with RNA-binding proteins (RBP), such as Nab2 or Ataxin-2, described in neural cells, ultimately reducing mRNA stability [94]. microRNAs can participate in the stabilization of mRNAs, which contributes strongly to their expression levels, by either recruiting certain RNA-binding proteins or reducing the activity of certain ribonucleases, increasing the availability of mRNA and supporting unaltered gene expression [92,95]. This mechanism could explain the lack of change in *FMR1* expression in gray zone carriers and the increase in *SMAD* expression. Since SMADs regulate GC proliferation, differentiation, and steroidogenesis [77,78], their enhanced expression could be due to interruptions in the aforementioned processes. While the main function of microRNAs has been described to be repressive, microRNAs can also increase gene expression. It can occur, as mentioned before, through stabilizing mRNA but also by reducing translational repression [80], activating transcription directly in the nucleus [96], or, working together with Argonaute proteins, increasing transcriptional activity by recruiting transcriptional activators or binding directly to the promotor region of their target gene [97]. Further experiments could investigate FMRP and *BMP*/*SMAD* expression levels at the protein level, observing whether the alterations in mRNA expression in gray zone patients influence translation. Additionally, in vitro experiments using a granulosa cell line without tumor cell properties, such as HGL5, could be performed to more accurately research the influence of miR-323a-3p on the BMP/SMAD pathway in a controlled environment.

Overall, the findings of our study demonstrate that, while carriers of gray zone alleles of the *FMR1* gene are generally considered phenotypically healthy, undeniable alterations in the transcriptional regulation process in ovarian cells are present. Importantly, for the first time, we have explored the relationship between *FMR1* and miR-323a-3p in the human ovary and observed that miR-323a-3p, known for its role in follicular development, is dysregulated in gray zone allele carriers, mediating diverse effects on the BMP/SMAD pathway in vitro and ex vivo. Additional research, focused on experiments at the protein level, is needed to further investigate the regulatory mechanism involving miR-323a-3p and its downstream targets, with the hope of better understanding the complexity of ovarian function and introducing novel therapeutic targets for diminished ovarian reserve.

## 4. Materials and Methods

### 4.1. Ethics Approval

This study was approved by the local ethics committee of the University of Heidelberg, Germany (permit no. S-602/2013, Amendments 2014, 2018, 2020, 2022). Prior to participating, all the patients signed an informed consent form.

### 4.2. Study Population

We prospectively enrolled 83 women undergoing IVF or IVF with intracytoplasmic sperm injection treatments from 2013 to 2023 at Heidelberg University Women’s Hospital. GC samples were obtained from all the participants. Only patients with sufficient retrieved material were included in this study, excluding patients with a history of autoimmune disease, a history of chemotherapy or radiation therapy, primary amenorrhea, and Turner Syndrome (45, X0 karyotype) or a known *FMR1*-premutation. Demographic and clinical data were collected by assessing the medical records of the patients and the questionnaires they answered. These data included their age, body mass index (BMI), baseline serum hormone levels (follicle stimulating hormone (FSH), luteinizing hormone (LH), estradiol (E2), and anti-Müllerian hormone (AMH)), and reproductive parameters (antral follicle count (AFC) and numbers of total retrieved and mature (MII) oocytes).

The patients were divided into two groups. In total, 55 women were presenting with a normal genotype. The remaining 28 participants were carriers of at least one gray zone, also called the intermediate length (41–54 CGG repeats) allele, and were assigned to the gray zone group. The individual experimental cohorts were sometimes adjusted (reduced in size) based on sample availability. The patients were split into five groups for general genotype analysis, defined by the CGG triplet repeat numbers in the alleles. In particular, the low number was defined as <26 CGG triplets, normal was defined as 26–34 CGG triplets, and high was defined as 35–54 CGG triplets [5]; this allowed for the distribution of the patients into five genotypes, namely low–low (LL, *n* = 7), low–normal (LN, *n* = 12), low–high (LH, *n* = 7), normal–normal (NN, *n* = 23), and normal–high (NH, *n* = 28).

### 4.3. Ovarian Stimulation and GC Collection

The patients were selected by their physicians independently of this study, and one of the appropriate protocols (either the long GnRH agonist or the GnRH antagonist protocol) was selected. The detailed procedures for ovarian stimulation and GC collection have been described previously [5,98].

### 4.4. Cell Culture

COV434 is a human ovarian granulosa tumor cell line derived from a solid primary tumor from a 27-year-old woman. This cell line is suitable for experimental studies of folliculogenesis because of its shared characteristics with proliferating GCs. COV434 cells maintain a stable 46, XX karyotype with minor chromosomal aberrations, can form intercellular connections, and can produce 17β-estradiol in the presence of FSH [99]. They also express *FMR1* and FMRP [18,71].

COV434 cells were cultured in Dulbecco’s modified Eagle’s medium (DMEM) with 20 mM glutamine (Biochrom GmbH, Berlin, Germany), supplemented with 10% heat-inactivated fetal bovine serum (Gibco, Life Technologies Corp., Darmstadt, Germany), at 37 °C under a 5% CO_2_ atmosphere [70]. The experiments were performed in triplicate under identical conditions.

### 4.5. RNA Extraction and Gene Expression Analysis

COV434 cells and patient GCs were suspended in RNAlater buffer and centrifuged at 5000× *g* for 5 min at room temperature, and the supernatant was removed. Total RNA was isolated using TRIzol (Life Technologies Corp., Carlsbad, CA, USA) and PEQGOLD PHA-SETRAP A 1.5 mL tubes (VWR International GmbH, Darmstadt, Germany), following the manufacturer’s protocols. The concentration and purity of the extracted RNA were determined using a NanoDrop 2000c UV-spectrometer (NanoDrop products, Wilmington, DE, USA).

For gene expression analysis, cDNA was synthesized by reverse transcribing 30 ng of total mRNA using oligo-(dT) primers and the M-MLV Reverse Transcriptase, RNase H Minus, Point Mutant (Promega, Madison, WI, USA). For microRNA expression analysis, specific cDNA was transcribed using the TaqMan MicroRNA Reverse Transcription Kit (Thermo Fisher Scientific #4366596, Waltham, MA, USA) and TaqMan MicroRNA Assay primers, following the manufacturer’s protocol.

In triplicate, real-time quantitative polymerase chain reaction (RT-qPCR) was performed using the Fast-7500 Real-Time PCR system (Applied Biosystems, Life Technologies, Carlsbad, CA, USA). cDNA from COV434 cells was used as a calibrator for gene mRNA expression level determination. For microRNA expression level determination, a patient from the control group, presenting with a normal genotype, was chosen randomly to serve as a comparative control (OR 399). No-template controls were used in every RT-qPCR plate as negative controls. Gene mRNA and microRNA levels were analyzed using the TaqMan Universal PCR Master Mix and TaqMan predesigned expression assays for *FMR1* (Hs00924544_m1), *BMPR2* (Hs00176148_m1), *SMAD1* (Hs00195432_m1), *SMAD3* (Hs00969210_m1), *SMAD4* (Hs00929647_m1), *SMAD5* (Hs00195437_m1), *SMAD9* (Hs00195441_m1), housekeeping gene TBP (Hs00427620_m1), housekeeping gene HPRT1 (Hs999909_m1), miR-323a-3p (477853), and endogenous control hsa-miR-16 (000391) [100,101], all purchased from Applied Biosystems (Life Technologies Corp., Carlsbad, CA, USA). The relative mRNA and microRNA expression levels were determined using the ∆∆Ct method [102], following the manufacturer’s instructions.

### 4.6. CGG Repeat Length Analysis (Adapted from *[103]*)

The CGG repeat lengths in the 5′ UTR of *FMR1* (NM_002024.5) exon 1 in the patients were analyzed using polymerase chain reaction (PCR) and an ALFexpress™ DNA sequencer (Amersham 1050; Pharmacia Biotech, Freiburg, Germany) or an ABI 3100/3130xl sequencer (Thermo Fisher Scientific Inc., Waltham, MA, USA), as described previously [5]. From May 2020 onwards, CGG repeat lengths have been analyzed using POI Triplet Repeat Primed Polymerase Chain Reaction (PCR, TP-PCR, and AmplideX^®^ PCR/CE FMR1 Kits; Asuragen Inc., Austin, TX, USA) as per the manufacturer’s protocol. The fragments were separated using a SeqStudio Genetic Analyzer (Thermo Fisher Scientific Inc.). GeneMapper™ v.5 software (Thermo Fisher Scientific Inc.) was used for electropherogram analysis.

### 4.7. Mimic Treatment

To investigate the direct effects of miR-323a-3p on *FMR1* and *SMAD* expression, COV434 cells were transfected with a specific miR-323a-3p mimic (Thermo Fisher Scientific #4464066, Waltham, MA, USA). Non-transfected cells and negative controls were used for comparison. The selected mimic concentration of 50 nM was based on previous studies [104], and the cells were exposed to the transfection mixture for 24 and 48 h.

The transfection mixture was prepared by mixing 250 μL of Opti-MEM | Reduced Serum Medium (#2193019, Gibco, Life Technologies Corp., Grand Island, NY, USA) with 7.5 μL of Lipofectamine 3000 (#2262699, Invitrogen, Life Technologies Corp., Carlsbad, CA, USA) and 10 μL of miR-323a-3p mimic (to achieve a final concentration of 50 nM/well). This mixture was added to a freshly trypsinized COV434 cell pellet (0.8 × 10^6^ cells/well) and seeded in a 6-well plate with 1750 μL of DMEM in each well. After 24 and 48 h, the cells were washed with 1 mL of phosphate-buffered saline, incubated with 25% trypsin solution at 37 °C under 5% CO_2_ for 5 min, resuspended in 1 mL DMEM, and collected. The cell pellet was obtained via centrifugation at 1700× *g* for 5 min at room temperature.

### 4.8. Statistical Analyses

Gene and microRNA level analyses were performed using the ∆∆Ct method [102]. Further, data were interpreted using GraphPad Prism software (Version 9.3.1), with statistical significance set at *p* < 0.05. The distribution of data was first determined using the Shapiro–Wilk test. Contingency analysis was performed with the χ^2^-test. Based on the results, pairwise comparisons were performed using either the unpaired Student’s *t*-test for datasets with a Gaussian distribution or the Mann–Whitney test for datasets that were not normally distributed.

## Figures and Tables

**Table 1:** BMI: body mass index; AFC: antral follicle count; FSH: follicle-stimulating hormone; LH: luteinizing hormone; AMH: anti-Müllerian hormone; MII oocytes: mature oocytes; SD: standard deviation. *p*-values represent significance between normal and gray zone genotype groups. No significant differences could be observed between the two groups.

Demographics	Normal Genotype		Gray Zone Genotype		*p*-Value
	*n*	Mean (±SD)	*n*	Mean (±SD)	
Age	55	34.47 (±4.91)	28	35.07 (±4.88)	0.6467
BMI	54	23.93 (±4.63)	28	24.24 (±4.63)	0.7736
FSH, U/L	48	9.07 (±3.68)	24	8.133 (±2.34)	0.2592
LH, U/L	52	5.39 (±2.31)	27	6.367 (±6.37)	0.1592
Estradiol, pg/nL	51	52.26 (±27.64)	26	59.03 (±58.27)	0.4905
AMH, ng/nL	51	2.10 (±2.03)	28	3.095 (±4.02)	0.1449
AFC	35	12.29 (±12.81)	18	10.22 (±9.25)	0.5475
Oocytes retrieved	55	7.77 (±5.74)	28	8.25 (±8.01)	0.7245
Mature (MII) oocytes	37	5.57 (±4.69)	17	6.588 (±5.82)	0.4949

## Data Availability

Data are available upon reasonable request from the first author, A.V.

## Supplementary Materials

The following supporting information can be downloaded at https://www.mdpi.com/article/10.3390/ijms26073192/s1.
