# Supplementary material for: Beyond Repetition: The Role of Gray Zone Alleles in the Upregulation of FMR1-Binding miR-323a-3p and the Modification of BMP/SMAD-Pathway Gene Expression in Human Granulosa Cells
Source: Int J Mol Sci. 2025 Mar 29;26(7):3192. doi: 10.3390/ijms26073192 (PMC11989689; doi:10.3390/ijms26073192)
Supplement: Supplementary file 1 [file ijms-26-03192-s001.zip › ijms-3488054-supplementary.pdf]

International Journal of
Molecular Sciences

MDPI

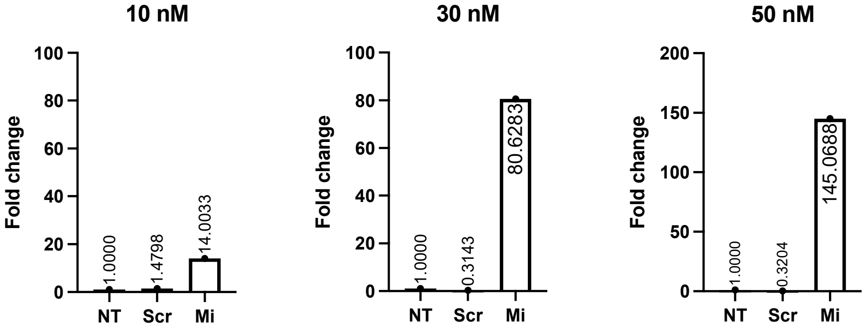

Supplementary Figure S1. Determination of the appropriate miR-323a-3p mimic concentration for successful transfection. Abbreviations: NT: non-transfected cells; Scr: scrambled siRNA treatment (negative control); Mi: mimic treatment. COV434 cells were treated with 10, 30, and 50 nM miR-323a-3p mimic, incubated for 24 h, and subsequently collected. The miRNA expression level was measured via TaqMan-based real-time polymerase chain reaction. miRNA levels were normalized to miR-16 levels, and the results are presented as comparative Ct (fold change) values (means ± SD; n = 3). miR-323a-3p expression was upregulated in all three treatments, with the 50 nM concentration showing the highest transfection efficiency (145-fold increase in miR-323a-3p levels compared to non-transfected cells).
